# Theme Trends and Knowledge-Relationship in Lifestyle Research: A Bibliometric Analysis

**DOI:** 10.3390/ijerph18147503

**Published:** 2021-07-14

**Authors:** Ah-Ram Kim, Hae Yean Park

**Affiliations:** 1 Department of Occupational Therapy, Graduate School, Yonsei University, Wonju 26493, Korea; aramkim495@gmail.com; 2 Department of Occupational Therapy, College of Software and Digital Healthcare Convergence, Yonsei University, Wonju 26493, Korea

**Keywords:** lifestyle, health, bibliometric analysis

## Abstract

Healthy living habits (healthy eating, regular physical activity, abstinence from smoking, restrictions on alcohol consumption, and stress management) can help prevent a significant number of diseases. The purpose of this study is to use a bibliometric analysis to analyze the relationships between countries, institutions and authors through lifestyle studies from 2016 to 2020 to find out the latest research trends. This study utilized bibliometric data collected through Scopus including thesis titles, authors, agencies, countries/regions, publication years, and keywords. Data were analyzed using the VOS viewer (Vers. 1.6.13; Leiden University, Leiden, The Netherlands) and the findings were used to visualize similarity mapping techniques. Publication of lifestyle-related research papers has steadily increased between 2016 and 2020. The country/region most actively conducting such research was the United States, also home to the majority of institutions conducting work in the field. PloS ONE published the most lifestyle-related research under the field of Medicine. Identified keywords were related to risk measures, psychosocial factors, prevention, health promotion, and risk factors. Lifestyle research is a promising field of research worldwide and has great potential to improve human health, the environment, and quality of life. The findings are expected to promote future research and give direction to the advancement of the field of research by comprehensively analyzing and summarizing lifestyle research trends.

## 1. Introduction

Worldwide, the rate of older adults (i.e., those aged 65 years and older) is expected to almost double over the next 30 years, from 12% to 22% [1]. This phenomenon will increase the rate of chronic diseases, considering that the prevalence of diseases increases with age and that older people are severely affected [2]. It will lead to higher social costs for health care and financial burdens for individuals and society. Therefore, an understanding of healthy aging is becoming increasingly necessary. Healthy living habits, including normal weight maintenance, smoking cessation, and regular exercise, contribute to reduced physical disability and mortality over time [3]. Such habits could help prevent a significant number of diseases [2].

The World Health Organization (WHO) defines lifestyle as “a specific type of behavior that can reduce disease and early death by personal, physical, mental, and social interaction”. According to the WHO, behavioral factors related to unhealthy lifestyles include a diet that lacks fruits and vegetables, smoking, lack of physical activity, a sedentary lifestyle, and drinking [4]. Lifestyle factors are multifaceted, interrelated, and related to multiple non-communicable diseases (NCDs) [5]. As of 2017, unhealthy behavior is estimated to account for more than 23 million deaths and 36.5% disability-adjusted life spans worldwide [6]. Additionally, a healthy lifestyle is the most effective strategy for preventing NCDs [7]. Therefore, understanding the association of studies on lifestyle factors is critical for establishing health policies and presenting a direction for future research. Many research institutes have supported policies to reduce the burden of disease by reducing unhealthy lifestyles [8,9].

Studies exploring trends in existing lifestyle-related research mainly used systematic reviews and meta-analysis with limited targets, such as specific age groups, specific diseases, and specific target groups [10,11,12,13]. Analyzing research trends using qualitative research methods has disadvantages such as limited time and funding and biased analysis results, depending on the researcher’s major. Therefore, literature research or content analysis is better suited for a micro-understanding of the details within a particular academic field [14,15].

The bibliometric analysis has been widely used in quantitative analysis of academic literature to describe the trends and contributions of countries/regions, journals, scholars, and keywords [16,17]. Co-occurrence word analysis is an important bibliometric technique from the late 1970s that can identify the main themes, investigate hot spots, and detect knowledge in the literature [18,19]. Thus, bibliometrics can contribute to monitoring the evolvement and patterns of effective publications [20]. In recent years, it has been applied to biomedicine and health care [21,22]. The bibliometric analysis provides researchers and related stakeholders with an opportunity to gain a beneficial understanding of the field of research and promote cross-disciplinary collaboration [23]. To achieve these benefits, such methods should also be applied to lifestyle-related research.

The purpose of our study is to examine the latest trends in lifestyle-themed studies using bibliometric analysis. This study is the first quantitative study to analyze research trends and knowledge relationships in lifestyle research. It will provide valuable guidance on future research directions in this rapidly evolving field.

## 2. Material and Methods

### 2.1. Data Collection

Scopus is an extensive international academic database containing authoritative information. It contains a variety of information available for bibliometric research, including the title of the paper, author, agency, country/region, year of publication, and keywords. It provides reliable data for bibliometric analysis in the field of recent lifestyle (or rehabilitation) research.

We searched for papers from 2016 to 2020 using the following search strategy: TITLE (lifestyle) AND DOCTYPE (ar) AND ACCESSYPE (OA) AND PUBYEAR < 2021. Two authors reviewed the resulting publications for the reliability of the search strategy. The study included all papers with an abstract, and excluded news, congresses, and letters to the editor. All data retrieved from the journal were organized in electronic spreadsheets.

### 2.2. Data Analysis and Visualization Maps

Our study aimed to leverage bibliometric analysis to identify bibliometric information, including knowledge structures in the field of lifestyle research, research boundaries, hot spots where research is actively conducted, and authors and institutions actively studying in related fields. Co-word analysis was used in each paper to compute the frequency of co-occurrences and perform hierarchical clustering based on co-occurrence information [18,19]. The VOS viewer (ver. 1.6.13; Leiden University, Leiden, The Netherlands) was used to extract bibliometric information about countries/regions, institutions, authors, and keywords. VOS viewer uses visualizations of similarity mapping techniques. It produces better-structured maps than other widely used techniques in the bibliometric field [24]. In particular, when constructing a map, the similarity is measured to represent the associated strength by the thickness or color of the line. Nearby items are items of high similarity, while items of low similarity are placed away from each other. Unlike other mapping programs, VOS viewer graphically represents them through bibliometric analysis in an easy-to-understand manner. Through network mapping, various maps were created on the simultaneous generation of countries/regions, institutions, authors, and keywords. Each node in the map is represented by a labeled circle. Larger nodes mean higher frequency, and smaller nodes mean lower frequency. The color of each circle is determined by the cluster to which each word belongs. The thickness and length of lines between nodes show the strength of the connectivity between the words.

### 2.3. Results Ethics

Data on bibliometric information were retrieved and downloaded from Scopus. This information is available to the public. Such data extraction does not involve direct contact or interaction with humans. Therefore, there is no ethical problem in research. Approval from the Research Ethics Committee is not required, including the use of these data.

## 3. Results

### 3.1. Publication Outputs

Based on our search strategy, we identified and incorporated 6075 publications on lifestyle from Scopus. The publication period was from 2016 to 2020, and it was only for journals with “lifestyle” included in the title. The number of annual publications in 2016, 2017, 2018, 2019, and 2020 was 1037, 1115, 1174, 1272, and 1477, respectively.

### 3.2. Distribution of Source Journals

Table 1 lists the top 10 journals on this topic. PloS ONE published the most papers (148/6075), followed by the International Journal of Environmental Research and Public Health (145/6075) and Nutrients (124/6075). The top 10 journals published 869 publications, accounting for 16.13% of all publications in this study.

### 3.3. Distribution and Co-Authorship of Countries/Regions

**Figure 1:**
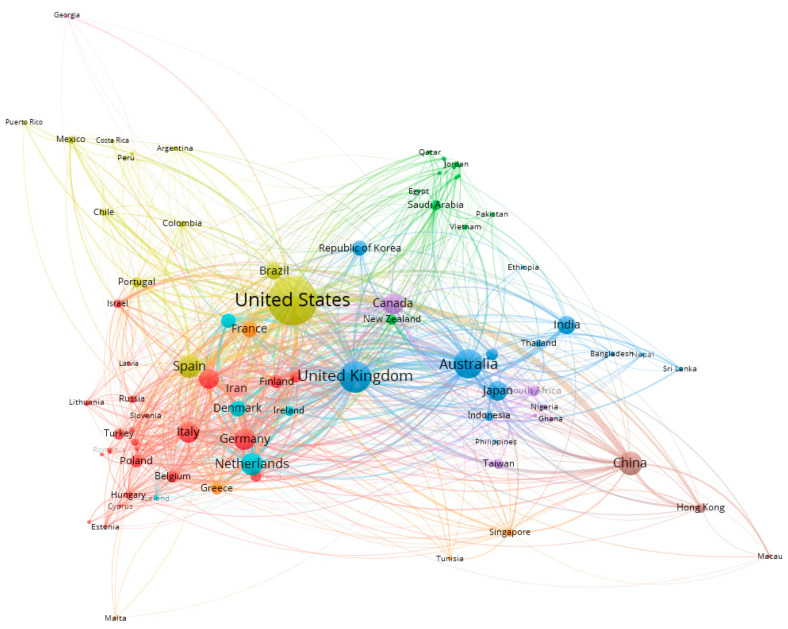
Distribution and co-authorship of countries/regions.

According to the search results, 6075 publications came from 225 countries/regions. As shown in Table 2, the United States has the largest number of publications (1586/6075) and the United Kingdom ranks second (674/6075), followed by Australia (573/6075). Figure 1 shows the location of the 225 countries/regions that were publishing lifestyle research. The co-authorship analysis of countries/regions reflects their relationship with the degree of collaboration in the field. The larger nodes represent more productive countries/regions in this field. The thickness and length of links between nodes represent the cooperative relationship between countries/regions. The 225 countries/regions from nine collaboration clusters are distinguished by different colors.

### 3.4. Distribution and Co-Authorship of Organizations

**Figure 2:**
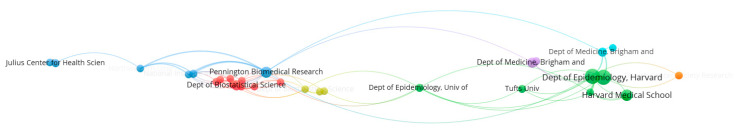
Distribution and co-authorship of organizations.

According to the search results, research organizations contributed to lifestyle research. Table 3 presents the top five most productive organizations in lifestyle research. Department of Nutrition, Harvard T.H. Chan School of Public Health, and Department of Epidemiology, Harvard T.H. Chan School of Public Health (20 publications) ranked first among all identified organizations, followed by the Tehran University (13 publications), the Harvard Medical School (12 publications) and the Pennington Biomedical Research Center (11 publications). Co-authorship analysis was performed by VOS viewer to display the visualization network map of organizations in lifestyle research. The link between institutions is determined by the number of publications co-authored between them, each of which published at least five papers and formed seven clusters. These clusters are shown in Figure 2.

### 3.5. Distribution and Co-Authorship of Authors

**Figure 3:**
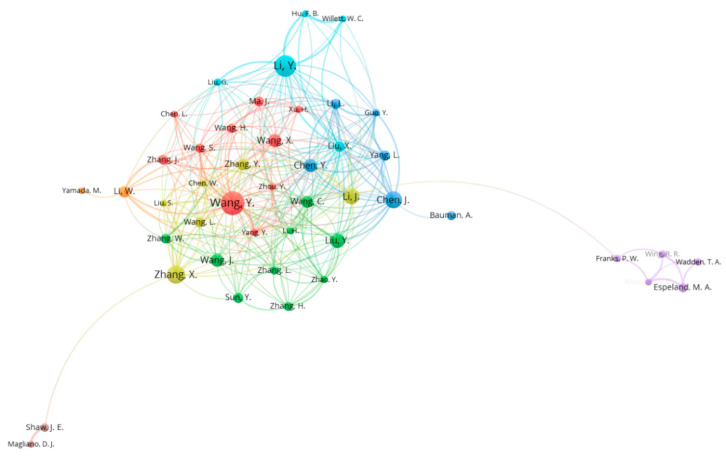
Distribution and co-authorship of authors.

According to the search results, lifestyle publications were written by authors. Table 4 presents the top 10 most productive authors in lifestyle research. Wang, Y (34 publications) ranked first among all authors, followed by Li, Y (31 publications) and Zhang, X. (26 publications). These clusters are shown in Figure 3.

### 3.6. Co-Occurrence Analysis of Top Keywords

**Figure 4:**
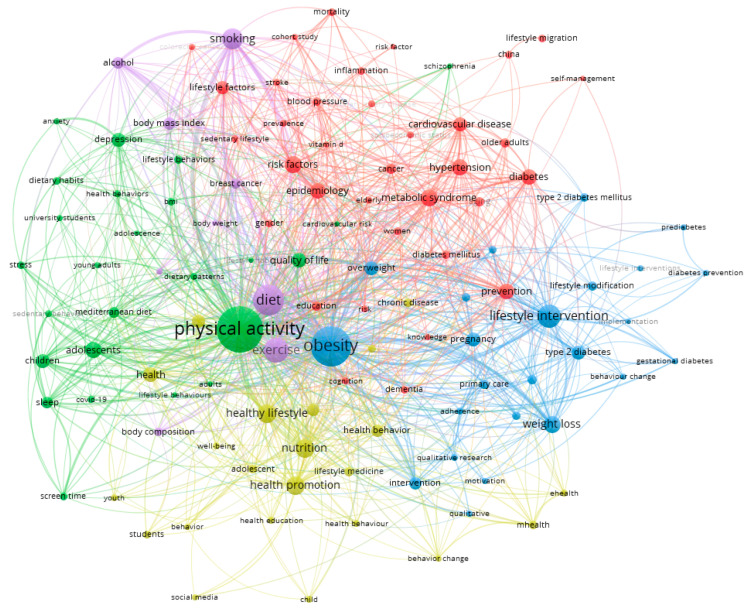
Co-occurrence analysis of top keywords.

We used VOS viewer to extract and cluster the top 100 keywords. The analysis was conducted after excluding the search term “lifestyle.” Appendix A shows the frequency and link strength of the top 100 keywords. As shown in Figure 4, we used VOS viewer to build a visualization network map of the 100 keywords in seven clusters with co-occurrence. The keywords physical activity (595), obesity (472), and diet (318) are located at the center of the visualization map. The node label is the keyword, and the node size represents its frequency. Links connecting two nodes represent a co-occurrence relationship between the keywords.

## 4. Discussion

This study collected and analyzed bibliometric information from lifestyle-related studies. The analysis identified research trends, countries, institutions, authors, and keywords related to lifestyle.

A change in the number of academic publications in a field is an important indicator of its evolutionary trend [25]. Lifestyle research has been on a steady rise over the past five years and has achieved remarkable results. It has been published over 500 times in the United States, the United Kingdom, and Australia. There is a large network of co-authors in various countries/regions who have shown cooperation internationally. Yellow represents mainly South American country/region, while red and sky blue included European country/region. The blue color indicates an Asian country/region, the brown color is a Chinese country/region, and the green color is a Middle Eastern country. These results are consistent with prior studies that suggest that cooperation between countries/regions may be affected by geographical proximity or common language [25]. This suggests the existence of a large gap between countries/regions. Through links with countries that lack research, the gap between public health should be bridged through the focus of preventive and population-based aims, treatment and patient-centered clinical practices.

All five organizations that are most actively researching lifestyle have been found to be American institutions. We show that the top institutions are consistent with the core countries of the study. These results show that the most productive countries and institutions are leading the trend in lifestyle research and have cooperative relationships. The arrangement of organs in Figure 2 is also horizontal. This shows that the related fields are mainly medical and the areas issued are limited. As lifestyle plays an important role not only in medical aspects of human life but also in other aspects, these results suggest a need for active multi-disciplinary research in various fields.

We identified authors conducting research in this field of study. Only 40 of them published more than 10 papers on lifestyle issues. These results confirm that there are many researchers interested in lifestyle, but cooperation and subsequent research among authors is limited. Collaboration among scholars promotes the flow of information and improves the efficiency of researchers by gradually reducing research costs [26]. Furthermore, encouraging collaboration between authors, agencies, and countries can increase the number of published authors and contribute to more effective research in relevant fields [25]. Therefore, it is desirable to strengthen cooperation between countries and authors worldwide for the diversity of lifestyle-related studies.

Keywords are standardized terms used to ensure that publications are indexed uniformly by topic. Mapping a co-keywords network by analyzing the frequency of co-keywords in several publications helped identify internal structures and trends in lifestyle research [27]. Analyzing the relationship between the top 100 keywords created five clusters. With respect to lifestyle characteristics, these five clusters were analyzed as follows.

Cluster 1 (red) mainly focused on risk diseases and included keywords such as metabolic syndrome, hypertension, diabetes, epidemiology, etc. Non-communicable diseases (NCDs), such as heart disease, stroke, cancer, chronic respiratory diseases, and diabetes, are the leading cause of mortality in the world [28]. NCDs are affected by lifestyle factors such as smoking, lack of diet, and lack of physical activity. This increases metabolic risk such as high blood pressure, dyslipidemia, glucose metabolic disorder, insulin resistance, or obesity [29]. Therefore, lifestyle interventions are needed to reduce the prevalence of major risk factors for chronic diseases and early detection. These efforts could significantly reduce their human and economic costs. For these efforts, academic research must be conducted continuously to accurately identify the risk factors of lifestyle.

Cluster 2 (green) focused primarily on relatively young subjects and psychosocial factors, including keywords such as adolescence, young, children, university students, anxiety, stress, and quality of life. According to a recent study, young adults and women are at higher risk of mental distress [30]. Additionally, a recent report by the Centers for Disease Control and Prevention (CDC) stated that young people in the U.S. (age 18–29 years) had the highest symptoms of mental pain distress. Lifestyle factors are a promising avenue for helpful treatments for depression and anxiety [31], as they affect our physical and mental health. It is necessary to study the living factors of depression and anxiety in the future based on a prior study [32,33] that found life mediation to be useful for preventing and treating mental diseases. These results confirmed that research on teenagers and young people as well as older adults is actively underway.

Cluster 3 (blue) focused on prevention and initial intervention and included keywords such as intervention, primary care, and prevention. NCDs are the leading causes of morbidity and mortality worldwide [34]. WHO [35] has made the prevention of NCDs a global priority. They generally have a long prodromal stage, taking many years to develop [34]. Lifestyle-related studies show that active research is being conducted as a preventive strategy that can slow down or stop the NCDs process. We confirm that lifestyle-related research should focus not only on disease management but also on disease prevention.

Cluster 4 (yellow) focuses on health promotion and includes keywords such as nutrition, health behavior, and behavior change. Countries around the world are implementing policies, strategies, and health programs to cope with the spread of chronic diseases and encourage healthy behavior. As the first step in prevention, the American College of Lifestyle Medicine (ACLM) introduced six ways to manage health through regular physical exercise, adequate and quality sleep, smoking cessation, stress management, and relationship maintenance. Lifestyle Medicine (LM) has produced significant changes in the concept of health, moving from a care-centered approach to an approach focused on promoting well-being [36].

Cluster 5 (purple) focuses on risk factors and includes keywords such as smoking, diet, alcohol, and body mass index (BMI). Unhealthy lifestyles include insufficient physical activity practices, adverse eating habits, sleep patterns, and alcohol and smoking [37,38]. Recently, a study found that very few Europeans achieved the recommended levels of physical activity, diet, low alcohol consumption, smoking cessation, and good sleep quality [39]. Therefore, our research supports the need to quit smoking, maintain a healthy weight, and abstain from drinking. Further research on risk factors is needed for a healthy lifestyle.

Our study is, to our knowledge, the first bibliometric analysis of lifestyle-related publications. Still, there are some limitations to this study. First, we selected most of the papers published in English (93.17%). Most of Scopus publications have been published in English, but there may be linguistic bias. Second, the quality of the papers published by Scopus is not uniform. This means that bibliometric analysis did not evaluate and analyze the quality of the paper, but only quantitative analysis. Third, the data currently used in this study, excluding search engine data such as PubMed, Google Scholar, and Web of Science, were analyzed only within the scope. Therefore, there is a possibility that a paper retrieved from a search engine other than Scopus may be missing. This requires further research to combine and analyze data from different search engines.

In terms of future research opportunities, we present future research directions through extensive reviews of literature using bibliometric analysis. Firstly, this study focuses on the term of “lifestyle” only. However, the field of lifestyle a diverse set of multifaceted. Thus, future research should focus on analyzing the concept from the lifestyle of various other disciplines of study. Secondly, given the nascent stage and the continuous rapid expansion of this field, it is quite evident that many more influential papers are to be witnessed. Thus, future research should continue to carry out such bibliometric studies on lifestyle within intervals of every five to seven years. This will contribute to the constant development of research concerning lifestyle.

## 5. Conclusions

This study uses bibliometric quantitative analysis and visualization network maps of data extracted from Scopus to show the research status, trends, co-country, co-authors, and co-keyword networks of lifestyles. We confirm that lifestyle research is a promising field of research worldwide and has great potential to improve human health and environment and improve quality of life. The findings are expected to promote direction for future research to advance the field of research by comprehensively analyzing and summarizing lifestyle research trends.

## Figures and Tables

**Table 1:** Top 10 journals publishing research on lifestyle research, 2016–2020.

Rank	Journal	Publisher	Country	Categories	Publication
1	PloS ONE	PUBLIC LIBRARY SCIENCE	United States	Multidisciplinary	148
2	International Journal of Environmental Research and Public Health	MDPI	Switzerland	Medicine	145
3	Nutrients	MDPI	Switzerland	Food Science, Nutrition	124
4	BMC Public Health	BMC	United Kingdom	Medicine	111
	BMJ Open	BMJ PUBLISHING GROUP			
5	Scientific Reports	NATURE RESEARCH	United Kingdom	Natural Science	79
6	American Journal of Lifestyle Medicine	SAGE PUBLICATIONS INC	United States	Lifestyle	78
7	Journal of Medical Internet Research	JMIR PUBLICATIONS, INC	Canada	Medicine	43
	Preventive Medicine	ACADEMIC PRESS INC ELSVIER SCIENCE	Netherlands	Preventive medicine, Public health	43
8	Sustainability	MDPI	Switzerland	Cross-disciplinary	36
9	Obesity	WILEY	United States	Endocrinology	33
10	Public Health Nutrition	CAMBRIDGE UNIV PRESS	United Kingdom	Nutrition	29

**Table 2:** Top 10 countries/regions publishing lifestyle research, 2016–2020.

Rank	Countries/Regions	Publication	Citation
1	United States	1586	14,324
2	United Kingdom	674	7159
3	Australia	573	4542
4	China	364	2654
5	Spain	349	3342
6	Netherlands	326	3549
7	Germany	314	3347
8	Italy	292	3144
9	Canada	277	2255
10	Sweden	271	3768
11	Japan	263	1176
12	India	217	845
13	Brazil	191	993
14	Iran	186	488
15	Denmark	165	1982
16	France	165	1753
17	South Korea	158	755
18	Norway	140	1238
19	Finland	127	1930
20	Poland	112	1245

**Table 3:** Top five organizations publishing lifestyle research, 2016–2020.

Rank	Organizations	Publication	Citation
1	Department on Nutrition, Harvard T.H. Chan School of Public Health	20	293
2	Department on Epidemiology, Harvard T.H. Chan School of Public Health	20	257
3	Tehran University	13	3
4	Harvard Medical School	12	89
5	Pennington Biomedical Research Center	11	133

**Table 4:** Top 10 most productive authors in lifestyle research, 2016–2020.

Rank	Author	Countries/Regions	Publication	Citation
1	Wang, Y.	China	34	159
2	Li, Y.	United States	31	716
3	Zhang, X.	United States	26	164
4	Chen, J.	China	24	215
5	Li, J.	United States	23	210
6	Liu, Y.	China	21	152
7	Wang, X.	China	19	139
8	Wang, J.	China	19	133
9	Chen, Y.	United Kingdom	19	126
10	Mercer, CH.	United Kingdom	17	176

## Data Availability

The data used in this analysis come from the Scopus and are available on its web page www.scopus.com (accessed on 26 January 2021).

## Appendix A

**Table A1:** Top 100 keywords in lifestyle research, 2016–2020.

Rank	Keyword	Frequency	Link Strength	Rank	Keyword	Frequency	Link Strength
1	Physical activity	595	538	51	Lifestyle change	41	27
2	Obesity	472	421	52	Type 2 diabetes mellitus	40	34
3	Diet	318	295	53	Chronic disease	40	33
4	Exercise	218	203	54	Dietary habits	39	34
5	Lifestyle intervention	203	175	55	Screen time	38	36
6	Smoking	170	157	56	Inflammation	38	33
7	Nutrition	144	137	57	Students	38	31
8	Health promotion	143	116	58	Gender	38	28
9	Healthy lifestyle	141	105	59	Lifestyle migration	38	7
10	Weight loss	124	116	60	Mortality	37	33
11	Metabolic syndrome	122	103	61	Stress	37	33
12	Risk factors	121	106	62	China	37	25
13	Adolescents	112	96	63	Dementia	36	33
14	Hypertension	111	94	64	Cardiovascular diseases	36	32
15	Diabetes	108	100	65	Insulin resistance	36	31
16	Prevention	106	100	66	BMI	35	32
17	Epidemiology	106	92	67	Women	35	28
18	Health	100	86	68	Socioeconomic status	34	32
19	Quality of life	98	86	69	Sedentary lifestyle	34	27
20	Cardiovascular disease	91	82	70	Dietary patterns	33	29
21	Overweight	89	85	71	Youth	33	27
22	Type 2 diabetes	89	78	72	Adherence	32	30
23	Pregnancy	89	77	73	Cognition	32	29
24	Children	88	81	74	Risk factors	32	26
25	Depression	87	80	75	Health behaviors	32	25
26	Lifestyle factors	83	48	76	Sedentary behaviors	31	30
27	Mental health	80	66	77	Qualitative research	31	27
28	Public health	79	54	78	COVID-19	31	26
29	Health behavior	77	64	79	Health behavior	31	24
30	Intervention	75	67	80	Coronary artery disease	31	24
31	Body mass index	75	64	81	Prevalence	30	27
32	Alcohol	70	65	82	Elderly	30	24
33	Sleep	65	64	83	eHealth	29	25
34	Mediterranean diet	59	55	84	Stroke	29	25
35	Cancer	58	56	85	Child	29	24
36	Breast cancer	56	47	86	Health education	29	24
37	Lifestyle modification	51	38	87	Well-being	29	21
38	Primary care	50	39	88	Behavior change	28	25
39	Older adults	50	35	89	Cohort study	28	25
40	Education	49	43	90	University students	28	23
41	Aging	48	42	91	Motivation	27	24
42	Randomized controlled trial	48	39	92	Behavior	27	23
43	Lifestyle medicine	47	21	93	Knowledge	27	23
44	Adolescent	45	41	94	Colorectal cancer	27	22
45	Lifestyle behaviors	44	36	95	Prediabetes	27	22
46	Childhood obesity	43	30	96	Anxiety	26	24
47	Blood pressure	42	38	97	Diabetes prevention	26	21
48	Diabetes mellitus	42	36	98	Lifestyle interventions	26	18
49	mHealth	41	40	99	Social media	26	16
50	Body composition	41	37	100	qualitative	25	23
